# 6,6′-Dimeth­oxy-2,2′-[2,2-dimethyl­propane-1,3-diylbis(nitrilo­methyl­idyne)]diphenol

**DOI:** 10.1107/S1600536808038002

**Published:** 2008-12-03

**Authors:** Chin Sing Yeap, Hadi Kargar, Reza Kia, Hoong-Kun Fun

**Affiliations:** a X-ray Crystallography Unit, School of Physics, Universiti Sains Malaysia, 11800 USM, Penang, Malaysia; b Department of Chemistry, School of Science, Payame Noor University (PNU), Ardakan, Yazd, Iran

## Abstract

The title Schiff base compound, C_21_H_26_N_2_O_4_, exhibits two crystallographically independent mol­ecules in the asymmetric unit with similar conformations. The imino groups are coplanar with the benzene rings; the maximum deviations of the N atoms from the planes comprising the benzene rings and the imino groups are −0.037 (4), 0.013 (4), −0.021 (5), and 0.008 (5) Å. The dihedral angles between the benzene rings in the two mol­ecules are 53.64 (17) and 51.93 (17)°. Strong intra­molecular O—H⋯N hydrogen bonds generate *S*(6) ring motifs. The N atoms are also in close proximity to the H atoms of the dimethyl­propane groups, with H⋯N distances between 2.54 and 2.75 Å. The crystal structure is further stabilized by weak inter­molecular C—H⋯O hydrogen bonds, weak inter­molecular C—H⋯π inter­actions and π–π contacts involving the imine C atom and two C atoms from the adjacent benzene rings.

## Related literature

For reference bond lengths, see Allen *et al.* (1987 ▶). For hydrogen-bond motifs, see: Bernstein *et al.* (1995 ▶). For information on Schiff base ligands and complexes and their applications, see: Calligaris & Randaccio (1987 ▶). For related structures, see: Li *et al.* (2005 ▶); Bomfim *et al.* (2005 ▶); Glidewell *et al.* (2006 ▶); Sun *et al.* (2004 ▶).
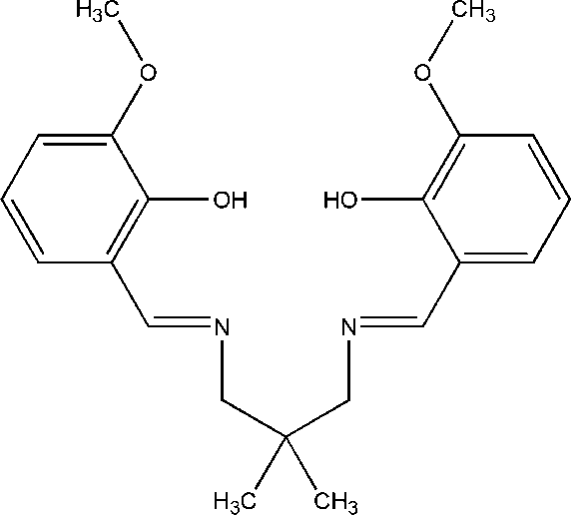


## Experimental

### 

#### Crystal data

C_21_H_26_N_2_O_4_
*M* *_r_* = 370.44
Monoclinic, 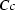

*a* = 6.8859 (3) Å
*b* = 30.8090 (14) Å
*c* = 18.8611 (9) Å
β = 96.102 (3)°
*V* = 3978.7 (3) Å^3^
*Z* = 8
Mo *K*α radiation
μ = 0.09 mm^−1^
*T* = 100.0 (1) K
0.45 × 0.38 × 0.26 mm

#### Data collection

Bruker SMART APEXII CCD area-detector diffractometer
Absorption correction: multi-scan (*SADABS*; Bruker, 2005 ▶) *T*_min_ = 0.962, *T*_max_ = 0.978
55368 measured reflections
7045 independent reflections
6422 reflections with *I* > 2σ(*I*)
*R*_int_ = 0.050

#### Refinement

*R*[*F* ^2^ > 2σ(*F* ^2^)] = 0.070
*wR*(*F* ^2^) = 0.202
*S* = 1.07
7045 reflections
495 parameters
2 restraints
H-atom parameters constrained
Δρ_max_ = 0.45 e Å^−3^
Δρ_min_ = −0.37 e Å^−3^

### 

Data collection: *APEX2* (Bruker, 2005 ▶); cell refinement: *APEX2*; data reduction: *SAINT* (Bruker, 2005 ▶); program(s) used to solve structure: *SHELXTL* (Sheldrick, 2008 ▶); program(s) used to refine structure: *SHELXTL*; molecular graphics: *SHELXTL*; software used to prepare material for publication: *SHELXTL* and *PLATON* (Spek, 2003 ▶).

## Supplementary Material

Crystal structure: contains datablocks global, I. DOI: 10.1107/S1600536808038002/zl2157sup1.cif

Structure factors: contains datablocks I. DOI: 10.1107/S1600536808038002/zl2157Isup2.hkl

Additional supplementary materials:  crystallographic information; 3D view; checkCIF report

## Figures and Tables

**Table 1:** Selected geometric parameters (Å) associated with π–π stacking interactions between the planar sections of C1*A*–C6*A*–C7*A*–N1*A* and C5*B*–C6*B*–C7*B*–N1*B*

C1*A*⋯C7*B*	3.298 (5)
C6*A*⋯C6*B*	3.300 (5)
C6*A*⋯C7*B*	3.250 (3)
C7*A*⋯C5*B*	3.393 (5)
C7*A*⋯C6*B*	3.267 (5)

**Table 2:** Hydrogen-bond geometry (Å, °)

*D*—H⋯*A*	*D*—H	H⋯*A*	*D*⋯*A*	*D*—H⋯*A*
O1*A*—H1*A*⋯N1*A*	0.84	1.82	2.570 (4)	148
O1*B*—H1*B*⋯N1*B*	0.84	1.85	2.581 (4)	144
O2*A*—H2*A*⋯N2*A*	0.84	1.89	2.625 (4)	145
O2*B*—H2*B*⋯N2*B*	0.84	1.87	2.607 (4)	146
C5*B*—H5*BA*⋯O1*B*^i^	0.95	2.59	3.233 (4)	125
C18*A*—H18*C*⋯N1*A*	0.98	2.56	2.900 (5)	100
C18*B*—H18*F*⋯N1*B*	0.98	2.54	2.889 (5)	101
C18*A*—H18*C*⋯N1*A*	0.98	2.56	2.900 (5)	100
C19*A*—H19*C*⋯N1*A*	0.98	2.71	3.021 (5)	99
C19*A*—H19*A*⋯N2*A*	0.98	2.63	2.950 (5)	99
C19*B*—H19*F*⋯N1*B*	0.98	2.70	3.012 (5)	99
C19*B*—H19*D*⋯N2*B*	0.98	2.63	2.946 (5)	99
C8*B*—H8*BA*⋯N2*B*	0.99	2.75	3.021 (5)	96
C10*A*—H10*B*⋯*Cg*1^ii^	0.99	2.73	3.481 (4)	133
C10*B*—H10*D*⋯*Cg*2^iii^	0.99	2.79	3.524 (4)	131

Symmetry codes: (i) 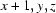; (ii) 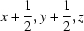; (iii) 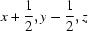. *Cg*1 and *Cg*2 are the centroids of the C12*B*–C17*B* and C12*A*–C17*A* benzene rings, respectively.

**Fig. 1.**
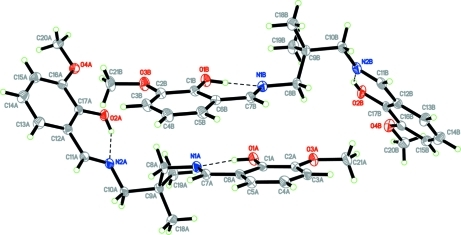
The molecular structure of the title compound with atom labels and 50% probability ellipsoids for non-H atoms. Dashed lines indicate intramolecular O—H···N hydrogen bonds.

**Fig. 2.**
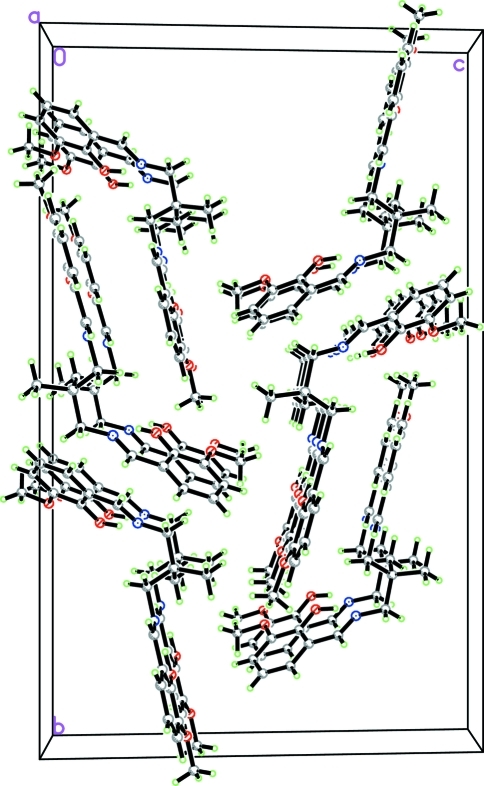
The crystal structure of the title compound, viewed down the a axis.

## supplementary crystallographic information

### Comment

Schiff bases are one of the most prevalent mixed-donor ligands in the field of coordination chemistry. They play an important role in the development of coordination chemistry related to catalysis and enzymatic reactions, magnetism, and supramolecular architectures (Calligaris & Randaccio, 1987). Structures of Schiff bases derived from substituted benzaldehydes and closely related to the title compound have been reported earlier (Li *et al.*, 2005; Bomfim *et al.*, 2005; Glidewell *et al.*, 2006; Sun *et al.*, 2004).

The molecule of the title compound (Fig. 1), consists of two crystallographically independent molecules, *A* and *B*, with similar conformations. The bond lengths (Allen *et al.*, 1987) and angles are within the normal ranges. The imino groups in both molecules are coplanar with the benzene rings they are attached to, and the dihedral angles between the benzene rings in molecules *A* and *B* are 53.64 (17) and 51.93 (17)°, respectively. Strong intramolecular O—H···N hydrogen bonds generate *S*(6) ring motifs (Bernstein *et al.*, 1995). The nitrogen atoms are also in close proximity to the hydrogen atoms of the dimethylpropane groups with H···N distances between 2.54 and 2.75 Å (Table 2). There is also a significant π-stacking interaction between the planar sections associated with C1A–C6A–C7A–N1A and C5B–C6B–C7B–N1B (Table 1), and the crystal structure is further stabilized by weak intermolecular C—H···O hydrogen bonds and weak intermolecular C—H···π interactions (Cg1 and Cg2 are the centroids of the C12B–C17B and C12A–C17A benzene rings) (Table 2).

### Experimental

In a 50 ml round-bottomed flask, 3-methoxy salicylaldehyde (2 mmol, 304 mg) was added into a 30 ml ethanolic solution of 2,2-dimethyl-1,3-propane diamine (1 mmol, 102 mg) and then the mixture was next refluxed for 1 h. The resulting yellow solid was filtered and washed with cold ethanol. Single crystals suitable for X-ray diffraction were obtained by slow evaporation of an ethanol solution at room temperature.

### Refinement

H atoms of the hydroxy groups were positioned by a freely rotating O—H bond and constrained with a fixed distance of 0.84 Å. The rest of the hydrogen atoms were positioned geometrically and refined using a riding model with C—H = 0.93–0.97 Å and U_iso_(H) = 1.2 or 1.5U_eq_(C). A rotating-group model was applied for the methyl hydrogen atoms of the methoxy groups. In the absence of sufficient anomalous scattering effects the Friedel pairs were merged prior to refinement.

### Crystal data

**Table:**

C_21_H_26_N_2_O_4_	*F*(000) = 1584
*M**_r_* = 370.44	*D*_x_ = 1.237 Mg m^−^^3^
Monoclinic, *C**c*	Mo *K*α radiation, λ = 0.71073 Å
Hall symbol: C -2yc	Cell parameters from 9913 reflections
*a* = 6.8859 (3) Å	θ = 2.5–31.8°
*b* = 30.8090 (14) Å	µ = 0.09 mm^−^^1^
*c* = 18.8611 (9) Å	*T* = 100 K
β = 96.102 (3)°	Block, yellow
*V* = 3978.7 (3) Å^3^	0.45 × 0.38 × 0.26 mm
*Z* = 8	

### Data collection

**Table:**

Bruker SMART APEXII CCD area-detector diffractometer	7045 independent reflections
Radiation source: fine-focus sealed tube	6422 reflections with *I* > 2σ(*I*)
graphite	*R*_int_ = 0.050
φ and ω scans	θ_max_ = 32.3°, θ_min_ = 1.3°
Absorption correction: multi-scan (*SADABS*; Bruker, 2005)	*h* = −10→10
*T*_min_ = 0.962, *T*_max_ = 0.978	*k* = −46→46
55368 measured reflections	*l* = −28→28

### Refinement

**Table:**

Refinement on *F*^2^	Primary atom site location: structure-invariant direct methods
Least-squares matrix: full	Secondary atom site location: difference Fourier map
*R*[*F*^2^ > 2σ(*F*^2^)] = 0.070	Hydrogen site location: inferred from neighbouring sites
*wR*(*F*^2^) = 0.202	H-atom parameters constrained
*S* = 1.07	*w* = 1/[σ^2^(*F*_o_^2^) + (0.1051*P*)^2^ + 8.0789*P*] where *P* = (*F*_o_^2^ + 2*F*_c_^2^)/3
7045 reflections	(Δ/σ)_max_ = 0.002
495 parameters	Δρ_max_ = 0.45 e Å^−^^3^
2 restraints	Δρ_min_ = −0.37 e Å^−^^3^

### Special details

**Table:**

Experimental. The low-temperature data was collected with the Oxford Cyrosystem Cobra low-temperature attachment.
Geometry. All esds (except the esd in the dihedral angle between two l.s. planes) are estimated using the full covariance matrix. The cell esds are taken into account individually in the estimation of esds in distances, angles and torsion angles; correlations between esds in cell parameters are only used when they are defined by crystal symmetry. An approximate (isotropic) treatment of cell esds is used for estimating esds involving l.s. planes.
Refinement. Refinement of F^2^ against ALL reflections. The weighted R-factor wR and goodness of fit S are based on F^2^, conventional R-factors R are based on F, with F set to zero for negative F^2^. The threshold expression of F^2^ > 2sigma(F^2^) is used only for calculating R-factors(gt) etc. and is not relevant to the choice of reflections for refinement. R-factors based on F^2^ are statistically about twice as large as those based on F, and R- factors based on ALL data will be even larger.

### Fractional atomic coordinates and isotropic or equivalent isotropic displacement parameters (Å^2^)

**Table:**

	*x*	*y*	*z*	*U*_iso_*/*U*_eq_	
O1A	0.6602 (3)	0.61681 (8)	0.79393 (15)	0.0220 (5)	
H1A	0.6514	0.6433	0.7837	0.033*	
O2A	0.1871 (4)	0.81687 (9)	0.62720 (15)	0.0262 (5)	
H2A	0.2552	0.8126	0.6662	0.039*	
O3A	0.7407 (4)	0.53607 (8)	0.82872 (15)	0.0231 (5)	
O4A	−0.0011 (4)	0.84221 (9)	0.50566 (15)	0.0256 (5)	
N1A	0.7730 (4)	0.69245 (9)	0.75795 (16)	0.0200 (5)	
N2A	0.5033 (4)	0.82012 (9)	0.71799 (16)	0.0209 (5)	
C1A	0.8478 (4)	0.60400 (10)	0.79486 (17)	0.0162 (5)	
C2A	0.8952 (5)	0.56063 (10)	0.81212 (17)	0.0187 (6)	
C3A	1.0852 (5)	0.54614 (11)	0.81211 (19)	0.0223 (6)	
H3AA	1.1163	0.5169	0.8243	0.027*	
C4A	1.2326 (5)	0.57441 (12)	0.7942 (2)	0.0228 (6)	
H4AA	1.3623	0.5641	0.7933	0.027*	
C5A	1.1880 (5)	0.61752 (11)	0.7780 (2)	0.0205 (6)	
H5AA	1.2879	0.6368	0.7666	0.025*	
C6A	0.9952 (5)	0.63275 (10)	0.77833 (17)	0.0175 (5)	
C7A	0.9487 (5)	0.67788 (10)	0.76016 (18)	0.0183 (5)	
H7AA	1.0504	0.6969	0.7497	0.022*	
C8A	0.7357 (5)	0.73797 (10)	0.74149 (19)	0.0210 (6)	
H8AA	0.6548	0.7404	0.6950	0.025*	
H8AB	0.8610	0.7530	0.7375	0.025*	
C9A	0.6290 (5)	0.76003 (10)	0.79997 (18)	0.0202 (6)	
C10A	0.6207 (5)	0.80896 (10)	0.78462 (19)	0.0213 (6)	
H10A	0.5659	0.8239	0.8245	0.026*	
H10B	0.7553	0.8199	0.7827	0.026*	
C11A	0.5777 (5)	0.84575 (10)	0.67483 (18)	0.0198 (6)	
H11A	0.7069	0.8561	0.6870	0.024*	
C12A	0.4712 (5)	0.85970 (11)	0.60758 (18)	0.0198 (6)	
C13A	0.5596 (5)	0.88882 (14)	0.5638 (2)	0.0267 (7)	
H13A	0.6866	0.8998	0.5783	0.032*	
C14A	0.4617 (6)	0.90153 (14)	0.4996 (2)	0.0285 (7)	
H14A	0.5225	0.9210	0.4698	0.034*	
C15A	0.2746 (6)	0.88615 (13)	0.4779 (2)	0.0253 (7)	
H15A	0.2095	0.8949	0.4333	0.030*	
C16A	0.1831 (5)	0.85812 (11)	0.52116 (19)	0.0206 (6)	
C17A	0.2814 (5)	0.84420 (10)	0.58698 (18)	0.0184 (5)	
C18A	0.7447 (7)	0.75372 (13)	0.8731 (2)	0.0300 (8)	
H18A	0.6757	0.7678	0.9097	0.045*	
H18B	0.8747	0.7667	0.8729	0.045*	
H18C	0.7580	0.7226	0.8835	0.045*	
C19A	0.4231 (6)	0.74167 (13)	0.7997 (2)	0.0289 (8)	
H19A	0.3512	0.7459	0.7525	0.043*	
H19B	0.3552	0.7567	0.8357	0.043*	
H19C	0.4306	0.7106	0.8108	0.043*	
C20A	−0.1043 (6)	0.85672 (13)	0.4401 (2)	0.0272 (7)	
H20A	−0.2355	0.8440	0.4346	0.041*	
H20B	−0.0335	0.8477	0.4002	0.041*	
H20C	−0.1146	0.8884	0.4406	0.041*	
C21A	0.7845 (7)	0.49164 (11)	0.8457 (2)	0.0291 (7)	
H21A	0.6651	0.4767	0.8560	0.044*	
H21B	0.8816	0.4902	0.8875	0.044*	
H21C	0.8368	0.4776	0.8051	0.044*	
O1B	0.5634 (3)	0.64558 (8)	0.58470 (15)	0.0216 (5)	
H1B	0.5639	0.6210	0.6041	0.032*	
O2B	0.2253 (4)	0.44378 (9)	0.76179 (15)	0.0255 (5)	
H2B	0.2786	0.4507	0.7254	0.038*	
O3B	0.6121 (4)	0.72611 (9)	0.54496 (16)	0.0260 (5)	
O4B	0.1066 (4)	0.41813 (9)	0.88280 (15)	0.0270 (5)	
N1B	0.7096 (4)	0.57124 (9)	0.62454 (16)	0.0204 (5)	
N2B	0.4884 (4)	0.44138 (9)	0.67216 (16)	0.0206 (5)	
C1B	0.7484 (4)	0.65956 (10)	0.58381 (18)	0.0170 (5)	
C2B	0.7791 (5)	0.70283 (11)	0.56300 (18)	0.0202 (6)	
C3B	0.9687 (5)	0.71851 (11)	0.5621 (2)	0.0228 (6)	
H3BA	0.9895	0.7476	0.5479	0.027*	
C4B	1.1290 (5)	0.69138 (12)	0.5821 (2)	0.0249 (7)	
H4BA	1.2578	0.7024	0.5821	0.030*	
C5B	1.1014 (5)	0.64898 (11)	0.6016 (2)	0.0215 (6)	
H5BA	1.2111	0.6308	0.6144	0.026*	
C6B	0.9113 (5)	0.63246 (11)	0.60275 (17)	0.0173 (5)	
C7B	0.8806 (5)	0.58763 (10)	0.62376 (18)	0.0187 (5)	
H7BA	0.9913	0.5699	0.6374	0.022*	
C8B	0.6908 (5)	0.52569 (11)	0.64410 (19)	0.0212 (6)	
H8BA	0.6393	0.5238	0.6911	0.025*	
H8BB	0.8211	0.5118	0.6483	0.025*	
C9B	0.5533 (5)	0.50155 (10)	0.58819 (18)	0.0194 (6)	
C10B	0.5641 (5)	0.45275 (10)	0.60503 (19)	0.0203 (6)	
H10C	0.4889	0.4367	0.5657	0.024*	
H10D	0.7019	0.4432	0.6071	0.024*	
C11B	0.5880 (5)	0.41536 (10)	0.71459 (19)	0.0208 (6)	
H11B	0.7098	0.4050	0.7021	0.025*	
C12B	0.5202 (5)	0.40099 (11)	0.78172 (18)	0.0193 (6)	
C13B	0.6361 (5)	0.37212 (13)	0.8259 (2)	0.0264 (7)	
H13B	0.7558	0.3617	0.8116	0.032*	
C14B	0.5756 (6)	0.35898 (14)	0.8901 (2)	0.0290 (7)	
H14B	0.6532	0.3393	0.9197	0.035*	
C15B	0.4011 (6)	0.37457 (12)	0.91147 (19)	0.0245 (7)	
H15B	0.3624	0.3658	0.9562	0.029*	
C16B	0.2833 (5)	0.40253 (11)	0.86886 (18)	0.0210 (6)	
C17B	0.3442 (5)	0.41619 (10)	0.80270 (18)	0.0191 (6)	
C18B	0.6217 (7)	0.50736 (13)	0.5136 (2)	0.0317 (8)	
H18D	0.5323	0.4919	0.4783	0.048*	
H18E	0.7537	0.4955	0.5134	0.048*	
H18F	0.6224	0.5383	0.5016	0.048*	
C19B	0.3445 (6)	0.51819 (13)	0.5896 (3)	0.0302 (8)	
H19D	0.3041	0.5142	0.6374	0.045*	
H19E	0.2564	0.5019	0.5550	0.045*	
H19F	0.3391	0.5491	0.5773	0.045*	
C20B	0.0398 (6)	0.40411 (14)	0.9482 (2)	0.0291 (7)	
H20D	−0.0861	0.4178	0.9538	0.044*	
H20E	0.1353	0.4124	0.9881	0.044*	
H20F	0.0245	0.3725	0.9474	0.044*	
C21B	0.6384 (6)	0.76900 (12)	0.5185 (3)	0.0313 (8)	
H21D	0.5114	0.7835	0.5103	0.047*	
H21E	0.6975	0.7673	0.4735	0.047*	
H21F	0.7242	0.7855	0.5535	0.047*	

### Atomic displacement parameters (Å^2^)

**Table:**

	*U*^11^	*U*^22^	*U*^33^	*U*^12^	*U*^13^	*U*^23^
O1A	0.0150 (10)	0.0177 (10)	0.0338 (13)	0.0015 (8)	0.0053 (9)	0.0003 (9)
O2A	0.0263 (13)	0.0208 (11)	0.0296 (13)	−0.0085 (10)	−0.0051 (10)	0.0080 (10)
O3A	0.0230 (11)	0.0157 (10)	0.0314 (13)	−0.0021 (9)	0.0057 (9)	0.0016 (9)
O4A	0.0227 (12)	0.0256 (12)	0.0266 (12)	−0.0056 (9)	−0.0064 (9)	0.0038 (10)
N1A	0.0208 (12)	0.0137 (11)	0.0250 (13)	0.0031 (9)	−0.0003 (10)	−0.0006 (10)
N2A	0.0233 (13)	0.0137 (11)	0.0244 (13)	0.0026 (10)	−0.0036 (10)	0.0004 (9)
C1A	0.0147 (12)	0.0152 (12)	0.0184 (13)	0.0005 (10)	−0.0007 (10)	−0.0028 (10)
C2A	0.0205 (14)	0.0159 (13)	0.0191 (13)	−0.0015 (10)	0.0001 (10)	−0.0013 (10)
C3A	0.0237 (15)	0.0164 (13)	0.0258 (15)	0.0036 (11)	−0.0020 (12)	−0.0026 (11)
C4A	0.0178 (13)	0.0207 (14)	0.0294 (16)	0.0031 (11)	−0.0002 (12)	−0.0036 (12)
C5A	0.0141 (12)	0.0186 (13)	0.0285 (16)	0.0013 (10)	0.0013 (11)	−0.0037 (11)
C6A	0.0151 (12)	0.0170 (13)	0.0200 (13)	−0.0003 (10)	−0.0007 (10)	−0.0027 (10)
C7A	0.0170 (13)	0.0156 (12)	0.0221 (14)	−0.0008 (10)	0.0010 (10)	−0.0008 (10)
C8A	0.0238 (14)	0.0124 (12)	0.0271 (15)	0.0032 (11)	0.0032 (12)	0.0015 (11)
C9A	0.0232 (14)	0.0154 (12)	0.0212 (14)	0.0015 (11)	−0.0005 (11)	−0.0012 (10)
C10A	0.0235 (15)	0.0128 (12)	0.0260 (15)	0.0014 (11)	−0.0055 (12)	−0.0003 (11)
C11A	0.0179 (13)	0.0173 (13)	0.0232 (14)	0.0011 (10)	−0.0022 (11)	0.0001 (11)
C12A	0.0175 (13)	0.0181 (13)	0.0234 (14)	−0.0004 (11)	0.0006 (11)	0.0006 (11)
C13A	0.0193 (15)	0.0313 (18)	0.0291 (17)	−0.0024 (13)	0.0010 (13)	0.0038 (14)
C14A	0.0268 (17)	0.0337 (19)	0.0258 (16)	−0.0005 (14)	0.0059 (13)	0.0058 (14)
C15A	0.0261 (16)	0.0268 (16)	0.0224 (15)	0.0063 (13)	−0.0002 (12)	0.0006 (12)
C16A	0.0214 (14)	0.0178 (13)	0.0217 (14)	0.0028 (11)	−0.0021 (11)	−0.0015 (11)
C17A	0.0180 (13)	0.0148 (12)	0.0221 (14)	0.0000 (10)	0.0002 (10)	−0.0007 (10)
C18A	0.043 (2)	0.0221 (15)	0.0228 (16)	0.0059 (15)	−0.0043 (15)	0.0005 (12)
C19A	0.0213 (15)	0.0220 (16)	0.043 (2)	−0.0010 (12)	0.0033 (14)	0.0057 (14)
C20A	0.0284 (17)	0.0277 (16)	0.0234 (16)	0.0010 (14)	−0.0066 (13)	0.0023 (13)
C21A	0.038 (2)	0.0159 (14)	0.0341 (19)	−0.0042 (14)	0.0081 (15)	−0.0020 (13)
O1B	0.0135 (10)	0.0181 (10)	0.0328 (13)	−0.0021 (8)	0.0010 (9)	0.0017 (9)
O2B	0.0310 (13)	0.0203 (11)	0.0270 (12)	0.0112 (10)	0.0106 (10)	0.0082 (9)
O3B	0.0221 (12)	0.0186 (11)	0.0368 (14)	0.0028 (9)	0.0014 (10)	0.0038 (10)
O4B	0.0273 (13)	0.0280 (13)	0.0277 (13)	0.0048 (10)	0.0125 (10)	0.0056 (10)
N1B	0.0221 (13)	0.0171 (12)	0.0216 (12)	−0.0021 (10)	0.0011 (10)	0.0012 (10)
N2B	0.0238 (13)	0.0144 (11)	0.0246 (13)	−0.0010 (10)	0.0077 (10)	0.0003 (10)
C1B	0.0144 (12)	0.0161 (12)	0.0205 (13)	−0.0006 (10)	0.0026 (10)	−0.0011 (10)
C2B	0.0201 (14)	0.0181 (13)	0.0222 (14)	−0.0006 (11)	0.0024 (11)	−0.0010 (11)
C3B	0.0218 (15)	0.0167 (13)	0.0302 (16)	−0.0024 (11)	0.0042 (12)	−0.0008 (12)
C4B	0.0211 (15)	0.0215 (15)	0.0326 (17)	−0.0014 (12)	0.0056 (13)	−0.0022 (13)
C5B	0.0164 (13)	0.0204 (14)	0.0278 (16)	−0.0022 (11)	0.0028 (11)	−0.0018 (12)
C6B	0.0168 (12)	0.0187 (13)	0.0164 (12)	−0.0002 (10)	0.0011 (10)	−0.0018 (10)
C7B	0.0185 (13)	0.0158 (12)	0.0217 (14)	0.0000 (10)	0.0013 (10)	−0.0013 (10)
C8B	0.0238 (15)	0.0142 (12)	0.0249 (15)	−0.0005 (11)	−0.0006 (12)	0.0030 (11)
C9B	0.0216 (14)	0.0154 (12)	0.0209 (14)	−0.0012 (10)	0.0016 (11)	0.0021 (10)
C10B	0.0244 (15)	0.0152 (12)	0.0226 (14)	−0.0020 (11)	0.0082 (12)	0.0005 (11)
C11B	0.0215 (14)	0.0156 (13)	0.0264 (15)	−0.0030 (11)	0.0075 (12)	−0.0014 (11)
C12B	0.0196 (14)	0.0163 (13)	0.0220 (14)	−0.0040 (10)	0.0017 (11)	0.0008 (10)
C13B	0.0188 (15)	0.0271 (16)	0.0328 (18)	0.0033 (12)	0.0009 (13)	0.0065 (14)
C14B	0.0232 (16)	0.0313 (18)	0.0315 (18)	−0.0018 (14)	−0.0026 (14)	0.0079 (14)
C15B	0.0260 (16)	0.0242 (15)	0.0226 (15)	−0.0061 (13)	−0.0005 (12)	0.0027 (12)
C16B	0.0244 (15)	0.0179 (13)	0.0209 (14)	−0.0020 (11)	0.0034 (11)	−0.0026 (11)
C17B	0.0234 (14)	0.0118 (11)	0.0223 (14)	−0.0022 (10)	0.0038 (11)	0.0011 (10)
C18B	0.047 (2)	0.0244 (17)	0.0244 (16)	−0.0090 (16)	0.0100 (16)	0.0029 (13)
C19B	0.0211 (16)	0.0275 (17)	0.041 (2)	−0.0001 (13)	−0.0013 (14)	0.0073 (15)
C20B	0.0352 (19)	0.0304 (18)	0.0233 (16)	−0.0035 (15)	0.0110 (14)	−0.0004 (13)
C21B	0.0323 (19)	0.0149 (14)	0.046 (2)	0.0030 (13)	0.0013 (17)	0.0026 (14)

### Geometric parameters (Å, °)

**Table:**

O1A—C1A	1.349 (4)	O1B—C1B	1.347 (4)
O1A—H1A	0.8400	O1B—H1B	0.8400
O2A—C17A	1.346 (4)	O2B—C17B	1.362 (4)
O2A—H2A	0.8400	O2B—H2B	0.8400
O3A—C2A	1.369 (4)	O3B—C2B	1.367 (4)
O3A—C21A	1.430 (4)	O3B—C21B	1.431 (5)
O4A—C16A	1.362 (4)	O4B—C16B	1.360 (4)
O4A—C20A	1.431 (4)	O4B—C20B	1.427 (5)
N1A—C7A	1.287 (4)	N1B—C7B	1.283 (4)
N1A—C8A	1.454 (4)	N1B—C8B	1.460 (4)
N2A—C11A	1.280 (5)	N2B—C11B	1.279 (4)
N2A—C10A	1.461 (4)	N2B—C10B	1.462 (4)
C1A—C2A	1.406 (4)	C1B—C2B	1.412 (4)
C1A—C6A	1.407 (5)	C1B—C6B	1.413 (4)
C2A—C3A	1.382 (5)	C2B—C3B	1.394 (5)
C3A—C4A	1.406 (5)	C3B—C4B	1.404 (5)
C3A—H3AA	0.9500	C3B—H3BA	0.9500
C4A—C5A	1.390 (5)	C4B—C5B	1.376 (5)
C4A—H4AA	0.9500	C4B—H4BA	0.9500
C5A—C6A	1.409 (4)	C5B—C6B	1.407 (5)
C5A—H5AA	0.9500	C5B—H5BA	0.9500
C6A—C7A	1.460 (4)	C6B—C7B	1.458 (5)
C7A—H7AA	0.9500	C7B—H7BA	0.9500
C8A—C9A	1.546 (5)	C8B—C9B	1.533 (5)
C8A—H8AA	0.9900	C8B—H8BA	0.9900
C8A—H8AB	0.9900	C8B—H8BB	0.9900
C9A—C19A	1.526 (5)	C9B—C19B	1.530 (5)
C9A—C18A	1.530 (5)	C9B—C10B	1.537 (4)
C9A—C10A	1.535 (4)	C9B—C18B	1.540 (5)
C10A—H10A	0.9900	C10B—H10C	0.9900
C10A—H10B	0.9900	C10B—H10D	0.9900
C11A—C12A	1.461 (4)	C11B—C12B	1.464 (5)
C11A—H11A	0.9500	C11B—H11B	0.9500
C12A—C13A	1.401 (5)	C12B—C17B	1.395 (5)
C12A—C17A	1.406 (5)	C12B—C13B	1.407 (5)
C13A—C14A	1.378 (5)	C13B—C14B	1.382 (6)
C13A—H13A	0.9500	C13B—H13B	0.9500
C14A—C15A	1.392 (6)	C14B—C15B	1.393 (6)
C14A—H14A	0.9500	C14B—H14B	0.9500
C15A—C16A	1.385 (5)	C15B—C16B	1.381 (5)
C15A—H15A	0.9500	C15B—H15B	0.9500
C16A—C17A	1.416 (4)	C16B—C17B	1.422 (5)
C18A—H18A	0.9800	C18B—H18D	0.9800
C18A—H18B	0.9800	C18B—H18E	0.9800
C18A—H18C	0.9800	C18B—H18F	0.9800
C19A—H19A	0.9800	C19B—H19D	0.9800
C19A—H19B	0.9800	C19B—H19E	0.9800
C19A—H19C	0.9800	C19B—H19F	0.9800
C20A—H20A	0.9800	C20B—H20D	0.9800
C20A—H20B	0.9800	C20B—H20E	0.9800
C20A—H20C	0.9800	C20B—H20F	0.9800
C21A—H21A	0.9800	C21B—H21D	0.9800
C21A—H21B	0.9800	C21B—H21E	0.9800
C21A—H21C	0.9800	C21B—H21F	0.9800
			
C1A···C7B	3.298 (5)	C7A···C5B	3.393 (5)
C6A···C6B	3.300 (5)	C7A···C6B	3.267 (5)
C6A···C7B	3.250 (3)		
			
C1A—O1A—H1A	109.5	C1B—O1B—H1B	109.5
C17A—O2A—H2A	109.5	C17B—O2B—H2B	109.5
C2A—O3A—C21A	115.4 (3)	C2B—O3B—C21B	115.9 (3)
C16A—O4A—C20A	116.0 (3)	C16B—O4B—C20B	115.9 (3)
C7A—N1A—C8A	119.2 (3)	C7B—N1B—C8B	119.2 (3)
C11A—N2A—C10A	118.0 (3)	C11B—N2B—C10B	118.4 (3)
O1A—C1A—C2A	118.7 (3)	O1B—C1B—C2B	118.3 (3)
O1A—C1A—C6A	121.7 (3)	O1B—C1B—C6B	122.3 (3)
C2A—C1A—C6A	119.6 (3)	C2B—C1B—C6B	119.4 (3)
O3A—C2A—C3A	125.5 (3)	O3B—C2B—C3B	125.4 (3)
O3A—C2A—C1A	114.3 (3)	O3B—C2B—C1B	114.7 (3)
C3A—C2A—C1A	120.2 (3)	C3B—C2B—C1B	119.9 (3)
C2A—C3A—C4A	120.5 (3)	C2B—C3B—C4B	120.1 (3)
C2A—C3A—H3AA	119.7	C2B—C3B—H3BA	120.0
C4A—C3A—H3AA	119.7	C4B—C3B—H3BA	120.0
C5A—C4A—C3A	119.8 (3)	C5B—C4B—C3B	120.7 (3)
C5A—C4A—H4AA	120.1	C5B—C4B—H4BA	119.7
C3A—C4A—H4AA	120.1	C3B—C4B—H4BA	119.7
C4A—C5A—C6A	120.2 (3)	C4B—C5B—C6B	120.2 (3)
C4A—C5A—H5AA	119.9	C4B—C5B—H5BA	119.9
C6A—C5A—H5AA	119.9	C6B—C5B—H5BA	119.9
C1A—C6A—C5A	119.6 (3)	C5B—C6B—C1B	119.8 (3)
C1A—C6A—C7A	120.4 (3)	C5B—C6B—C7B	120.6 (3)
C5A—C6A—C7A	120.0 (3)	C1B—C6B—C7B	119.6 (3)
N1A—C7A—C6A	121.5 (3)	N1B—C7B—C6B	122.4 (3)
N1A—C7A—H7AA	119.3	N1B—C7B—H7BA	118.8
C6A—C7A—H7AA	119.3	C6B—C7B—H7BA	118.8
N1A—C8A—C9A	111.0 (3)	N1B—C8B—C9B	110.9 (3)
N1A—C8A—H8AA	109.4	N1B—C8B—H8BA	109.5
C9A—C8A—H8AA	109.4	C9B—C8B—H8BA	109.5
N1A—C8A—H8AB	109.4	N1B—C8B—H8BB	109.5
C9A—C8A—H8AB	109.4	C9B—C8B—H8BB	109.5
H8AA—C8A—H8AB	108.0	H8BA—C8B—H8BB	108.0
C19A—C9A—C18A	110.5 (3)	C19B—C9B—C8B	109.8 (3)
C19A—C9A—C10A	110.3 (3)	C19B—C9B—C10B	110.4 (3)
C18A—C9A—C10A	107.5 (3)	C8B—C9B—C10B	108.7 (3)
C19A—C9A—C8A	110.3 (3)	C19B—C9B—C18B	110.8 (3)
C18A—C9A—C8A	110.2 (3)	C8B—C9B—C18B	110.2 (3)
C10A—C9A—C8A	108.0 (3)	C10B—C9B—C18B	106.8 (3)
N2A—C10A—C9A	113.5 (3)	N2B—C10B—C9B	113.6 (3)
N2A—C10A—H10A	108.9	N2B—C10B—H10C	108.9
C9A—C10A—H10A	108.9	C9B—C10B—H10C	108.9
N2A—C10A—H10B	108.9	N2B—C10B—H10D	108.9
C9A—C10A—H10B	108.9	C9B—C10B—H10D	108.9
H10A—C10A—H10B	107.7	H10C—C10B—H10D	107.7
N2A—C11A—C12A	122.4 (3)	N2B—C11B—C12B	122.1 (3)
N2A—C11A—H11A	118.8	N2B—C11B—H11B	118.9
C12A—C11A—H11A	118.8	C12B—C11B—H11B	118.9
C13A—C12A—C17A	120.2 (3)	C17B—C12B—C13B	119.9 (3)
C13A—C12A—C11A	119.4 (3)	C17B—C12B—C11B	120.7 (3)
C17A—C12A—C11A	120.4 (3)	C13B—C12B—C11B	119.4 (3)
C14A—C13A—C12A	119.8 (3)	C14B—C13B—C12B	119.9 (4)
C14A—C13A—H13A	120.1	C14B—C13B—H13B	120.0
C12A—C13A—H13A	120.1	C12B—C13B—H13B	120.0
C13A—C14A—C15A	120.8 (4)	C13B—C14B—C15B	120.1 (4)
C13A—C14A—H14A	119.6	C13B—C14B—H14B	119.9
C15A—C14A—H14A	119.6	C15B—C14B—H14B	119.9
C16A—C15A—C14A	120.3 (3)	C16B—C15B—C14B	121.2 (3)
C16A—C15A—H15A	119.9	C16B—C15B—H15B	119.4
C14A—C15A—H15A	119.9	C14B—C15B—H15B	119.4
O4A—C16A—C15A	124.7 (3)	O4B—C16B—C15B	126.2 (3)
O4A—C16A—C17A	115.3 (3)	O4B—C16B—C17B	114.7 (3)
C15A—C16A—C17A	120.0 (3)	C15B—C16B—C17B	119.1 (3)
O2A—C17A—C12A	123.1 (3)	O2B—C17B—C12B	122.5 (3)
O2A—C17A—C16A	118.0 (3)	O2B—C17B—C16B	117.8 (3)
C12A—C17A—C16A	118.9 (3)	C12B—C17B—C16B	119.7 (3)
C9A—C18A—H18A	109.5	C9B—C18B—H18D	109.5
C9A—C18A—H18B	109.5	C9B—C18B—H18E	109.5
H18A—C18A—H18B	109.5	H18D—C18B—H18E	109.5
C9A—C18A—H18C	109.5	C9B—C18B—H18F	109.5
H18A—C18A—H18C	109.5	H18D—C18B—H18F	109.5
H18B—C18A—H18C	109.5	H18E—C18B—H18F	109.5
C9A—C19A—H19A	109.5	C9B—C19B—H19D	109.5
C9A—C19A—H19B	109.5	C9B—C19B—H19E	109.5
H19A—C19A—H19B	109.5	H19D—C19B—H19E	109.5
C9A—C19A—H19C	109.5	C9B—C19B—H19F	109.5
H19A—C19A—H19C	109.5	H19D—C19B—H19F	109.5
H19B—C19A—H19C	109.5	H19E—C19B—H19F	109.5
O4A—C20A—H20A	109.5	O4B—C20B—H20D	109.5
O4A—C20A—H20B	109.5	O4B—C20B—H20E	109.5
H20A—C20A—H20B	109.5	H20D—C20B—H20E	109.5
O4A—C20A—H20C	109.5	O4B—C20B—H20F	109.5
H20A—C20A—H20C	109.5	H20D—C20B—H20F	109.5
H20B—C20A—H20C	109.5	H20E—C20B—H20F	109.5
O3A—C21A—H21A	109.5	O3B—C21B—H21D	109.5
O3A—C21A—H21B	109.5	O3B—C21B—H21E	109.5
H21A—C21A—H21B	109.5	H21D—C21B—H21E	109.5
O3A—C21A—H21C	109.5	O3B—C21B—H21F	109.5
H21A—C21A—H21C	109.5	H21D—C21B—H21F	109.5
H21B—C21A—H21C	109.5	H21E—C21B—H21F	109.5
			
C21A—O3A—C2A—C3A	−1.4 (5)	C21B—O3B—C2B—C3B	4.6 (5)
C21A—O3A—C2A—C1A	179.3 (3)	C21B—O3B—C2B—C1B	−175.4 (3)
O1A—C1A—C2A—O3A	−2.0 (4)	O1B—C1B—C2B—O3B	−0.6 (4)
C6A—C1A—C2A—O3A	178.6 (3)	C6B—C1B—C2B—O3B	179.3 (3)
O1A—C1A—C2A—C3A	178.6 (3)	O1B—C1B—C2B—C3B	179.4 (3)
C6A—C1A—C2A—C3A	−0.7 (5)	C6B—C1B—C2B—C3B	−0.7 (5)
O3A—C2A—C3A—C4A	−179.9 (3)	O3B—C2B—C3B—C4B	179.8 (3)
C1A—C2A—C3A—C4A	−0.6 (5)	C1B—C2B—C3B—C4B	−0.2 (5)
C2A—C3A—C4A—C5A	1.4 (5)	C2B—C3B—C4B—C5B	1.0 (6)
C3A—C4A—C5A—C6A	−1.0 (5)	C3B—C4B—C5B—C6B	−0.9 (6)
O1A—C1A—C6A—C5A	−178.1 (3)	C4B—C5B—C6B—C1B	−0.1 (5)
C2A—C1A—C6A—C5A	1.2 (5)	C4B—C5B—C6B—C7B	−179.7 (3)
O1A—C1A—C6A—C7A	0.6 (5)	O1B—C1B—C6B—C5B	−179.3 (3)
C2A—C1A—C6A—C7A	179.9 (3)	C2B—C1B—C6B—C5B	0.9 (5)
C4A—C5A—C6A—C1A	−0.3 (5)	O1B—C1B—C6B—C7B	0.3 (5)
C4A—C5A—C6A—C7A	−179.1 (3)	C2B—C1B—C6B—C7B	−179.5 (3)
C8A—N1A—C7A—C6A	178.3 (3)	C8B—N1B—C7B—C6B	178.1 (3)
C1A—C6A—C7A—N1A	−0.8 (5)	C5B—C6B—C7B—N1B	−179.1 (3)
C5A—C6A—C7A—N1A	177.9 (3)	C1B—C6B—C7B—N1B	1.3 (5)
C7A—N1A—C8A—C9A	−125.4 (3)	C7B—N1B—C8B—C9B	−128.7 (3)
N1A—C8A—C9A—C19A	−67.3 (4)	N1B—C8B—C9B—C19B	−67.9 (4)
N1A—C8A—C9A—C18A	55.0 (4)	N1B—C8B—C9B—C10B	171.2 (3)
N1A—C8A—C9A—C10A	172.1 (3)	N1B—C8B—C9B—C18B	54.4 (4)
C11A—N2A—C10A—C9A	−130.8 (3)	C11B—N2B—C10B—C9B	−134.3 (3)
C19A—C9A—C10A—N2A	−55.8 (4)	C19B—C9B—C10B—N2B	−54.7 (4)
C18A—C9A—C10A—N2A	−176.4 (3)	C8B—C9B—C10B—N2B	65.8 (4)
C8A—C9A—C10A—N2A	64.8 (4)	C18B—C9B—C10B—N2B	−175.3 (3)
C10A—N2A—C11A—C12A	−178.6 (3)	C10B—N2B—C11B—C12B	−177.8 (3)
N2A—C11A—C12A—C13A	178.2 (3)	N2B—C11B—C12B—C17B	−1.5 (5)
N2A—C11A—C12A—C17A	−1.6 (5)	N2B—C11B—C12B—C13B	179.7 (3)
C17A—C12A—C13A—C14A	−1.4 (6)	C17B—C12B—C13B—C14B	−0.2 (6)
C11A—C12A—C13A—C14A	178.8 (4)	C11B—C12B—C13B—C14B	178.7 (3)
C12A—C13A—C14A—C15A	0.7 (6)	C12B—C13B—C14B—C15B	−0.6 (6)
C13A—C14A—C15A—C16A	0.7 (6)	C13B—C14B—C15B—C16B	1.4 (6)
C20A—O4A—C16A—C15A	−1.0 (5)	C20B—O4B—C16B—C15B	0.4 (5)
C20A—O4A—C16A—C17A	178.6 (3)	C20B—O4B—C16B—C17B	178.6 (3)
C14A—C15A—C16A—O4A	178.2 (4)	C14B—C15B—C16B—O4B	176.8 (4)
C14A—C15A—C16A—C17A	−1.4 (5)	C14B—C15B—C16B—C17B	−1.3 (5)
C13A—C12A—C17A—O2A	−178.7 (3)	C13B—C12B—C17B—O2B	−179.3 (3)
C11A—C12A—C17A—O2A	1.1 (5)	C11B—C12B—C17B—O2B	1.8 (5)
C13A—C12A—C17A—C16A	0.7 (5)	C13B—C12B—C17B—C16B	0.2 (5)
C11A—C12A—C17A—C16A	−179.5 (3)	C11B—C12B—C17B—C16B	−178.6 (3)
O4A—C16A—C17A—O2A	0.5 (5)	O4B—C16B—C17B—O2B	1.7 (4)
C15A—C16A—C17A—O2A	−179.8 (3)	C15B—C16B—C17B—O2B	−180.0 (3)
O4A—C16A—C17A—C12A	−179.0 (3)	O4B—C16B—C17B—C12B	−177.8 (3)
C15A—C16A—C17A—C12A	0.7 (5)	C15B—C16B—C17B—C12B	0.5 (5)

### Hydrogen-bond geometry (Å, °)

**Table:**

*D*—H···*A*	*D*—H	H···*A*	*D*···*A*	*D*—H···*A*
O1A—H1A···N1A	0.84	1.82	2.570 (4)	148
O1B—H1B···N1B	0.84	1.85	2.581 (4)	144
O2A—H2A···N2A	0.84	1.89	2.625 (4)	145
O2B—H2B···N2B	0.84	1.87	2.607 (4)	146
C5B—H5BA···O1B^i^	0.95	2.59	3.233 (4)	125
C18A—H18C···N1A	0.98	2.56	2.900 (5)	100
C18B—H18F···N1B	0.98	2.54	2.889 (5)	101
C18A—H18C···N1A	0.98	2.56	2.900 (5)	101
C19A—H19C···N1A	0.98	2.71	3.021 (5)	99
C19A—H19A···N2A	0.98	2.63	2.950 (5)	100
C19B—H19F···N1B	0.98	2.70	3.012 (5)	99
C19B—H19D···N2B	0.98	2.63	2.946 (5)	99
C8B—H8BA···N2B	0.99	2.75	3.021 (5)	96
C10A—H10B···Cg1^ii^	0.99	2.73	3.481 (4)	133
C10B—H10D···Cg2^iii^	0.99	2.79	3.524 (4)	131

Symmetry codes: (i) *x*+1, *y*, *z*; (ii) *x*+1/2, *y*+1/2, *z*; (iii) *x*+1/2, *y*−1/2, *z*.
